# Global karst springs hydrograph dataset for research and management of the world’s fastest-flowing groundwater

**DOI:** 10.1038/s41597-019-0346-5

**Published:** 2020-02-20

**Authors:** Tunde Olarinoye, Tom Gleeson, Vera Marx, Stefan Seeger, Rouhollah Adinehvand, Vincenzo Allocca, Bartolome Andreo, James Apaéstegui, Christophe Apolit, Bruno Arfib, Augusto Auler, Juan Antonio Barberá, Christelle Batiot-Guilhe, Timothy Bechtel, Stephane Binet, Daniel Bittner, Matej Blatnik, Terry Bolger, Pascal Brunet, Jean-Baptiste Charlier, Zhao Chen, Gabriele Chiogna, Gemma Coxon, Pantaleone De Vita, Joanna Doummar, Jannis Epting, Matthieu Fournier, Nico Goldscheider, John Gunn, Fang Guo, Jean Loup Guyot, Nicholas Howden, Peter Huggenberger, Brian Hunt, Pierre-Yves Jeannin, Guanghui Jiang, Greg Jones, Herve Jourde, Ivo Karmann, Oliver Koit, Jannes Kordilla, David Labat, Bernard Ladouche, Isabella Serena Liso, Zaihua Liu, Nicolas Massei, Naomi Mazzilli, Matías Mudarra, Mario Parise, Junbing Pu, Nataša Ravbar, Liz Hidalgo Sanchez, Antonio Santo, Martin Sauter, Vianney Sivelle, Rannveig Øvrevik Skoglund, Zoran Stevanovic, Cameron Wood, Stephen Worthington, Andreas Hartmann

**Affiliations:** 1 Chair of Hydrological Modeling and Water Resources, University of Freiburg, 79098 Freiburg, Germany; 2 Department of Civil Engineering, University of Victoria, Victoria, Canada; 3 Chair of Hydrology, University of Freiburg, 79098 Freiburg, Germany; 4 Department of Earth Sciences, Shiraz University, Shiraz, Iran; 5 Dipartimento di Scienze della Terra, dell’Ambiente e delle Risorse, University of Naples Federico II, Napoli, Italy; 6 Department of Geology and Centre of Hydrogeology of the University of Málaga, Málaga, Spain; 7 Instituto Geofísico del Perú, Lima, Peru; 8 Universidad Nacional Agraria La Molina, Maestria en Recursos Hídricos, Lima, Perú; 9 Parc Naturel Régional des Grands Causses (PRNGC), Saint-Léons, France; 10 Aix Marseille Univ, CNRS, IRD, INRA, Coll France, CEREGE, Aix-en-Provence, France; 11 Instituto do Carste/Carste Ciência e Meio Ambiente, Belo Horizonte, Brazil; 12 HydroSciences Montpellier (HSM), Univ. Montpellier, CNRS, IRD, Montpellier, France; 13 Earth and Environment, Franklin and Marshall College, Lancaster, Pennsylvania, USA; 14 ISTO, Université d’Orléans, CNRS, BRGM, OSUC, Orléans, France; 15 Faculty of Civil, Geo and Environmental Engineering, Technical University of Munich, Arcisstr. 21, 80333 Munich, Germany; 16 ZRC SAZU Karst Research Institute, Postojna, Slovenia; 17 Cave and Karst Specialist, Vientiane, Laos; 18 BRGM, Univ. Montpellier, Montpellier, France; 19 Institute of Applied Geosciences, Karlsruhe Institute of Technology (KIT), Kaiserstr. 12, 76131 Karlsruhe, Germany; 20 Institute for Geography, University of Innsbruck, Innrain 52, Innsbruck, Austria; 21 School of Geographical Sciences, University of Bristol, Bristol, BS8 1SS UK; 22 Department of Geology, American University of Beirut, Beirut, Lebanon; 23 Applied and Environmental Geology, Department of Environmental Sciences, University of Basel, Bernoullistr. 32, 4056 Basel, Switzerland; 24 Normandie Univ, UNIROUEN, UNICAEN, CNRS, M2C, 76000 Rouen, France; 25 School of Geography, Earth and Environmental Science, University of Birmingham, Birmingham, UK; 26 Institute of Karst Geology, Chinese Academy of Geological Sciences, Guilin, China; 27 GET Laboratory, Toulouse University/CNRS/IRD, Toulouse, France; 28 Department of Civil Engineering, University of Bristol, Bristol, UK; 29 Barton Springs/Edward Aquifer Conservation District, Austin, Texas USA; 30 Institut Suisse de Spéléologie et de Karstologie, ISSKA, CH-2301 La Chaux-de-Fonds, Switzerland; 31 Department for Environment and Water, Government of South Australia, Adelaide, Australia; 32 University of São Paulo, São Paulo, Brazil; 33 Institute of Ecology, School of Natural Sciences and Health, Tallinn University, Uus-Sadama 5, 10120 Tallinn, Estonia; 34 Department of Applied Geology, Georg-August-University Göttingen, Goldschmidstr. 3, 37077 Göttingen, Germany; 35 Géosciences Environnement Toulouse (GET) - CNRS – UPS – IRD – CNES, 14 avenue Edouard Belin, 31400 Toulouse, France; 36 Department of Earth and Environmental Sciences, University Aldo Moro, Bari, Italy; 37 INRAE, Avignon Université, EMMAH, F-84000 Avignon, France; 38 LOCEAN Laboratory, Sorbonne-Université/CNRS/IRD/ MNHN, Paris, France; 39 University Federico II, Naples, Italy; 40 Department of Geography, University of Bergen, Bergen, Norway; 41 Faculty for Mining and Geology, University of Belgrade, Belgrade, Serbia; 42 Worthington Groundwater, Ontario, Canada

**Keywords:** Hydrology, Water resources

## Abstract

Karst aquifers provide drinking water for 10% of the world’s population, support agriculture, groundwater-dependent activities, and ecosystems. These aquifers are characterised by complex groundwater-flow systems, hence, they are extremely vulnerable and protecting them requires an in-depth understanding of the systems. Poor data accessibility has limited advances in karst research and realistic representation of karst processes in large-scale hydrological studies. In this study, we present World Karst Spring hydrograph (WoKaS) database, a community-wide effort to improve data accessibility. WoKaS is the first global karst springs discharge database with over 400 spring observations collected from articles, hydrological databases and researchers. The dataset’s coverage compares to the global distribution of carbonate rocks with some bias towards the latitudes of more developed countries. WoKaS database will ensure easy access to a large-sample of good quality datasets suitable for a wide range of applications: comparative studies, trend analysis and model evaluation. This database will largely contribute to research advancement in karst hydrology, supports karst groundwater management, and promotes international and interdisciplinary collaborations.

## Abstract (summary)

**Table:** 

Measurement(s)	hydrographic feature • fluid flow rate
Technology Type(s)	digital curation
Factor Type(s)	geographic location • year
Sample Characteristic - Environment	karst • spring • groundwater

Machine-accessible metadata file describing the reported data: 10.6084/m9.figshare.11336507

## Background & Summary

Karst aquifers are essential sources of drinking water to about 10% of the world’s population^1^. In many regions across the globe, karst groundwater is also an indispensable resource for ecosystems, agriculture and, economic activities, as well as for tourism and recreation^2,3^. For example, in Europe, 21.6% of the land surface is underlain by carbonate rock^4^ which contributes up to 50% of supplied drinking water in some countries^5–7^. However, groundwater flow in karst aquifers is characterised by a complex interplay of fast-flowing conduit and slow-flowing matrix systems^8,9^. Hence, the storage capacity of karst aquifers is variable and systems are extremely vulnerable to climatic pressures, human impacts and contamination^10^. In order to ensure adequate protection of karst water sources, in-depth hydrogeological knowledge is necessary.

Large-scale modelling and comparative water resource research have shown the great value of large datasets in hydrology^11^. Numerous studies have applied these large datasets for several purposes such as model evaluation, global parameter estimations, impact studies, statistical and comparative analyses. For instance, large-scale hydrological models such as WaterGAP^12^ used discharge data from the Global Runoff Data Centre (https://www.bafg.de/GRDC) for parameter estimation. Likewise, streamflow data from the Model Parameter Estimation Experiment (MOPEX)^13^ and the Global Runoff Data Centre (GDRC) were combined to derive global base flow indexes and recession constants^14^. Streamflow observations of near-natural catchments obtained from UNESCO’s European Water Archive (EWA) were used to investigate the streamflow trends across Europe and differentiated the impacts from climatic variability and anthropogenic drivers^15^. The same dataset was also applied to assess the sensitivity of streamflow to storage changes in Europe^16^.

Even though the existing large hydrological databases (e.g. MOPEX, GRDC) have brought great advances to the understanding of hydrological processes and their simulation, these databases do not explicitly consider karst areas as karst spring discharges are under-represented. Access to data has been identified as a major impediment in quantifying karstification, modelling flow dynamics and transport processes of karst groundwater^2,17^. Studies involving large-scale parameter estimation or comparative studies in karst hydrology are still fairly rare and unrealistic representations of hydrological processes in karst regions can still be found in many large-scale hydrological models^17,18^. The need to advance research in karst hydrology especially on larger spatial scales, combined with the importance and peculiarities of karst aquifers, therefore requires a consolidated, global database for karst systems. Recent advances in providing large-scale information on karst aquifers include the development of World Karst Aquifer Map (WOKAM)^4,19^, which is the first to accurately map karst regions worldwide, or the SNO KARST database^20^ that provides long-term observations of hydro meteorological and geochemical variables of several karst observatories across France, including karst spring discharge. The SNO KARST also offers a new tools for characterizing and modelling flow in karst aquifers^21^ or assess their vulnerability^22^.

With the WoKaS database, we provide the first known effort to create a global database of karst spring discharge observations. It is the result of an intense and global effort to make a large number of karst spring hydrographs accessible for karst researchers and the wider hydrological communities. Data from individual researchers and research networks like the Karst Commission of the International Association of Hydrogeologists (IAH) was combined with karst spring hydrographs from national databases and digitized spring discharge data from scientific publications. Access to WoKaS database will motivate large scale and comparative karst hydrology studies, help to improve representation of karstic processes in large-scale models, improve management of karst groundwater, and will promote international and interdisciplinary collaborations. We encourage future users of the datasets to contact researcher or agency that provided the datasets to start a fruitful research collaboration.

## Methods

**Fig. 1:**
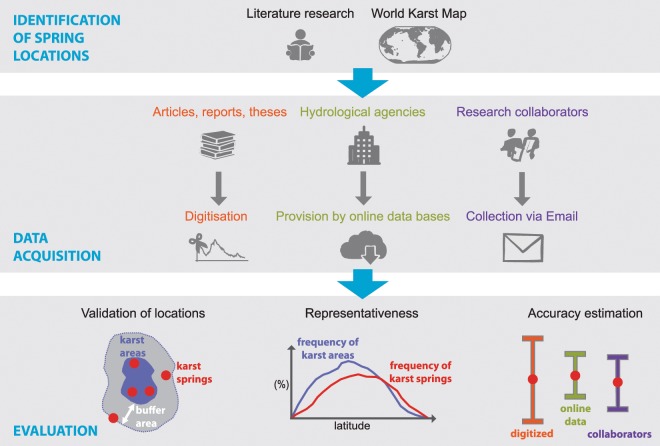
Workflow of the karst spring discharge observation database development.

The development of the WoKaS database followed three steps: (i) identification of karst spring locations across the globe; (ii) sourcing for discharge observations of the identified springs; and (iii) evaluation of collected datasets, which included technical validation and quality assessment. The workflow of these steps is illustrated in Fig. 1.

### Identifying karst spring locations

Firstly, we assembled the list of karst springs in countries with carbonate outcrops identified from the World Karst Aquifer Map^4,19^. For each country with carbonate outcrops, we performed an extensive literature search with a set of keywords consisting of: (1) country’s name; (2) karst; (3) spring; and (4) hydrology. From all the identified materials (articles, conference proceedings, reports, theses, news bulletins, books), we extracted karst spring names, location coordinates, elevation as well as land cover, catchment area (km^2^), defined as the topographic boundary within which the spring is located, recharge area (km^2^), defined as the area contributing to the recharge of the aquifer drained by the spring, and factors influencing discharge if such information were available. Several spring locations were also collected from WOKAM, which provides a list of relevant karst springs for each country, and from reviewing national databases.

### Data acquisition

Time series of karst spring discharge observations were collected from three sources: (1) published data including scientific articles, reports and theses; (2) project partners and collaborators; and (3) public databases of national hydrological services. For each source, the method for data extraction, collection and gathering were different.

### Published articles, reports and theses

A web search routine protocol was developed to look-up all publications in karst and non-karst hydrology containing karst spring hydrographs. Firstly, karst spring hydrographs of identified locations (see previous subsection) were searched in published journal articles. The keyword “hydrograph” was added to the set of keywords used in location identification (country’s name, karst, spring and hydrology). Occasionally, the country’s name was substituted with the spring’s name for a more specific web search. The search was further extended to published reports from NGOs, government agencies and PhD theses. The web search protocols for karst spring hydrographs and location identification were similar, hence, the two processes were usually run concurrently.

To extract the spring discharge observations from the published articles, theses and reports, we used *WebPlotDigitizer* (https://github.com/ankitrohatgi/WebPlotDigitizer). WebPlotDigitizer is an open source, web-based, semi-automatic digitization tool developed with HTML5 that works on most common web browsers. The hydrographs were cut out from the original publications, saved as image or pdf files and imported to WebPlotDigitizer. The raw discharge values for the total duration of the observation period were then extracted. Python codes for daily time step interpolation were used to post-process the extracted raw values.

Spring discharge observation time step are not usually stated in publications. Therefore, when the temporal resolution of the observation was unknown, the interpolation time step used was irregular and dependent on the resolution of the extracted figure: plot quality, number of plotted variables, and length or duration of the hydrograph. For instance, hydrographs that covered longer time periods only show seasonal and annual events, hence, a discharge variability could only be captured on monthly time steps. Whereas, if the observation period was shorter, individual events could be identified and discharge values could be extracted on a daily temporal resolution.

### Research partners and collaborators

Additional data were acquired through the karst research community. Calls for data contribution were made at conferences, through social media platforms (Twitter and Facebook) and emails soliciting data support for the database to various research commissions, institutes, working groups and researchers with relevant datasets.

### Hydrological agencies

A large number of karst spring discharge observations were obtained from national hydrological services that provide online access to their datasets. In total, we collected discharge datasets from ten national databases mainly in Europe and the United States of America (Table 1). Most of this data is in the public domain or published under the creative commons (CC-BY) license and could be directly combined with the data obtained from other sources (see above). Data from databases (Banque Hydro, eHYD, LUBW and NRFA) that do not provide their discharge data under the open data or CC-BY license are made available only as annual averages in the data repository^23^. To access this data at daily resolution, we provide an automatic download routine written with R, which extracts the karst spring discharge time series from the databases’ webpages directly. In addition, the download procedure updates the spring discharge time series of all databases in case new observations were added after publication of the WoKaS database (see following subsection).

**Table 1:** Hydrological databases where datasets were downloaded. If automatic download is “Yes” the corresponding database is included in the automatic download routine; see Hydrological agencies subsection. All databases were last accessed in September 2019.

Country	Database/agency name	Database access	Automatic download
France	Ministère de l’Écologie, du Développement Durable et de l’Énergie (BANQUE HYDRO)	http://www.hydro.eaufrance.fr/indexd.php	Yes
France	SNO KARST, OSU OREME	https://data.oreme.org/observation/snokarst	No
Austria	Bundesministerium für Nachhaltigkeit und Tourismus (eHYD)	https://ehyd.gv.at/	Yes
Germany	Bayerisches Landesamt für Umwelt	https://www.gkd.bayern.de/de/grundwasser/quellschuettung//tabellen	Yes
Germany	Landesanstalt für Umwelt Baden-Württemberg (LUBW)	http://udo.lubw.baden-wuerttemberg.de/public/pages/selector/index.xhtml	Yes
Ireland	Environmental Protection Agency (EPA HydroNet)	http://www.epa.ie/hydronet/#Groundwater	Yes
Slovenia	Agencija Republike Slovenije za okolje (ARSO)	http://vode.arso.gov.si/hidarhiv/pov_arhiv_tab.php	Yes
US	U.S. Geological Survey, National Water Information System (USGS)	https://waterdata.usgs.gov/nwis/uv/?referred_module=gw	Yes
UK	Nation River Flow Archive (NRFA)	https://nrfa.ceh.ac.uk/data/search	Yes
Croatia	Croatian Meteorological and Hydrological Service (DHMZ)	http://hidro.dhz.hr/	No

## Data Records

**Fig. 2:**
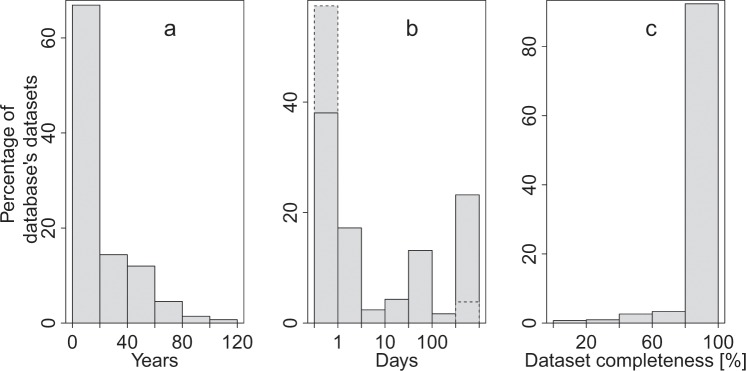
Properties of the collected datasets in the WoKaS database. (**a**) Time span of spring discharge observations, (**b**) temporal resolution of spring discharge observations with “Days” axis plotted on a Log scale (dashed-line bars indicate the shift in the percentage of datasets with <  = 1 day and a year temporal resolution if the time series from databases that do not hold open data or CC-BY license are replaced with higher resolution time series datasets obtainable through the automatic download routine; see subsection Hydrological Agencies), (**c**) completeness of discharge datasets.

The WoKaS database includes over 400 karst spring discharge observations from more than 30 countries across the globe covering a wide range of hydrologic and climatic diversity. The datasets which are freely available for download^23^ are accessible in comma-separated values (CSV) file format. Time series datasets cover time spans ranging from a couple of months to a maximum of 120 years (Fig. 2a). Over 60% of the dataset is made by discharge time series observations of up to 20 years and within this subset, over 90% of the time series have discharge observations greater than a year, more than 65% cover an observation periods greater than 5 years and above 35% have more than 10 years of discharge observations. More than 30% have time series measurements of > 20 years (Fig. 2a). Considering all collected datasets with those from databases without CC-BY license, which are available as annual averages^23^ (see hydrological agencies subsection), ca. 40% of the datasets have temporal resolution of ≤1 day and above 20% have a year resolution (Fig. 2b). If the datasets from databases without CC-BY license are substituted by higher resolution time series data which are accessible through the download routine (see hydrological agencies subsection), the percentage of datasets with ≤1 day temporal resolution increased to almost 60%. Subsequently, datasets with a year temporal resolution are reduced by 20% (Fig. 2b). Dataset completeness describes the percentage of total discharge observations of a dataset without missing values. More than 90% of the datasets in the WoKaS database are gap-free for the obtained hydrograph duration (Fig. 2c).

### Accuracy and quality of datasets

The accuracy and quality of the datasets were defined based on four criteria (Table 2): (1) if the discharge observation measurement is known, (2) recognition of individual events on the spring’s hydrograph, (3) recognition of seasonal events on the spring’s hydrograph and (4) identification of recession events on the hydrograph. These criteria (mostly based on the data source - see subsection data acquisition) were used to assign five quality classes, from A (very high) to C3 (very low). Generally, datasets from hydrological databases, research partners and collaborators fall within the quality class A or B. Since digitized datasets were extracted from hydrograph plots, inaccuracies were inherited from quality and observation length of the hydrograph plot that was to be digitized. For example, when discharge observation covered several years, only seasonal variability was visually identifiable and individual discharge events could not be recognised. Meanwhile, both seasonal variability and individual events are visually recognisable for discharge observations extending over fewer years (<5years). Consequently, the digitized datasets are sub-divided to class C1 (individual events identifiable, recession periods recognizable), C2 (individual events identifiable, recession periods not clearly recognizable) and C3 (individual events not identifiable, recession periods not clearly recognizable).

**Table 2:** Datasets quality description. The symbol “✓” indicates that the corresponding requirement is fulfilled and “✗” indicates that the requirement is not fulfilled.

Class	Description	Criteria			
		Measurement type known	Individual event	Seasonality	Individual recession
A	Very High	✓	✓	✓	✓
B	High	✗	✓	✓	✓
C1	Medium (digitized < = 5 years)	✗	✓	✓	Recognisable
C2	Low (digitized > 5 years)	✗	✗	✓	Recognisable
C3	Very Low (unclear, poor plot)	✗	✗	✓	✗

**Fig. 3:**
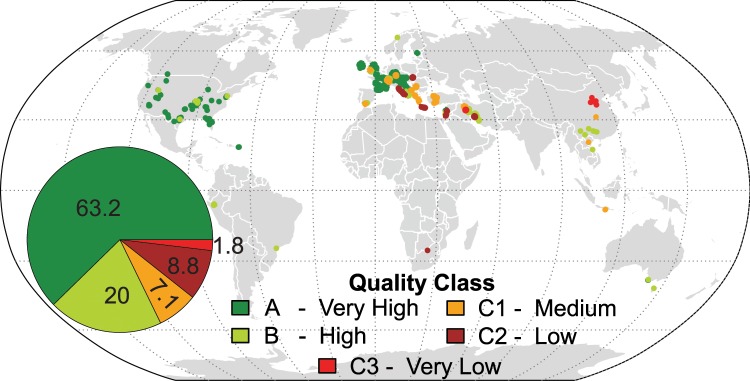
Distribution of WoKaS datasets based on assigned quality classes. The coloured points on the map are WoKaS locations, attributed colour codes correspond to the quality class. Numbers shown on pie chart in the map are percentage distribution of WokaS datasets based on defined quality classes.

Based on the defined quality classes, a high percentage of WoKaS datasets are of good quality (Fig. 3), approximately 62% and 20% of the datasets are of class A and B respectively (Fig. 3). Class A datasets are predominantly found in the northern hemisphere between the latitudes of 30° and 60°, in the same region in which we have 80% of the WoKaS datasets (see subsection spatial representativeness of datasets). Similarly, class B datasets are distributed within these latitudes in Europe and Asia, and also in Australia. The class C datasets are found in the Middle East, Asia and Southern Africa, these are the regions where spring discharge datasets have been digitised from publications due to the scarcity or unavailability of direct spring discharge observations.

## Technical Validation

The collected datasets were evaluated by: (1) determining the accuracy of the identified spring locations with respect to carbonate rock outcrop of WOKAM in order to exclude non-karstic springs and (2) determining the spatial representativeness of the database for karst areas by comparing the distribution of the identified locations over all latitudes with the distribution of carbonate rock outcrops over the world’s land surface.

### Accuracy of identified locations

A fundamental prerequisite for a spring to be considered karstic is that, it must be fed by a karst aquifer. The recharge area of karst aquifer can lie exclusively (autogenic recharge) or partially (allogenic recharge) within carbonate rock areas^9^. In some cases, recharge of karst aquifers is partly due to groundwater flow coming from adjoining aquifers, such as the alluvial ones. Also, karst spring outlets exist within the carbonate rock formation but seldom, a karst aquifer-fed spring may have its discharge outlet in a non-carbonate formation. All collected spring locations were compared with the carbonate rock areas indicated by WOKAM^19^ to ensure that they fall within the carbonate rock outcrop. Taking into account karst aquifer-fed springs outside the carbonate rock outcrop, we allowed for a buffer zone around the carbonate rock areas. We used this simplified strategy because detailed, site-specific field information was not obtainable for the large set of collected karst spring locations. We defined the buffer width by the maximum distance of spring locations provided by WOKAM from the WOKAM carbonate rock areas (17.2 km). We consider this buffer distance reasonable as the WOKAM developers could rely on local experts that could confirm the karstic characteristics of all the included spring locations. Using this procedure, over 90% of the identified spring locations fall on the carbonate rock outcrops and approximately 5% are within the buffer area.

### Spatial representativeness of datasets

**Fig. 4:**
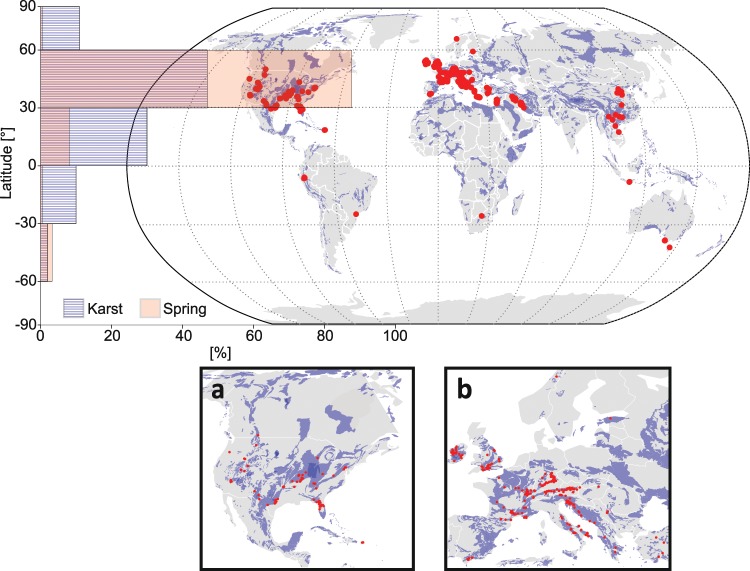
Global coverage of karst spring discharge observation datasets. Red points on the globe represent WoKaS spring locations and blue areas are the carbonate rock outcrops from WOKAM. The frequencies of WoKaS spring and carbonate rock area distributions across the latitudes are respectively represented by the transparent red and blue bars on the horizontal histogram. Maps insert below are zoom plots of North America (**a**) and Europe (**b**).

Likewise karst landscape areas, karst springs are not evenly distributed globally. Consequently, it is important to ensure that the WoKaS database is representative of karst’s landscape distribution. Therefore, we compared the frequency of karst areas over all latitudes with the frequency of spring locations over the same latitudes. Using 30° grid steps, we found that the distribution of karst areas resembles the distribution of WoKaS spring locations (Fig. 4) but with a considerable bias towards the wealthier and data-rich regions of Europe and North America. At those latitudes (30°N–60°N), we found approximately 50% of the total global karst area and 80% of the WoKaS datasets. More (financial) resources and attention have been directed towards hydrological studies and monitoring in these regions, which is a common and well-known problem of the global representativeness of experimental hydrology^25^. We expect that future experimental works and research collaborations will allow for compensating this imbalances. In some regions, notably the Middle East and China, information relating to hydrological monitoring are considered confidential and only few authorized people can have access to them. We hope that more open data policies will improve access to this data in the future to increase the benefits of scientific exchange for both the research communities and societies.

## Usage Notes

### Data repository organisation

WoKaS is a compilation of volumetric karst spring discharge observation datasets from different sources (see subsection data acquisition). The data repository^23^ holds a single packaged zip archive named “WoKaS_Data_Record”, which contains a “read_me” text file that provides guide information for users and four subfolders: WoKaS_Hydrograph_Metafile, WoKaS_Hydrograph_Datasets, Auto_Download_Routine and WoKaS_Summary_Plot. The contents of the files and subfolders contained in the zip archive are described below:

(A) WoKaS_Hydrograph_Metafile contains a xlsx file “WoKaS_Metafile” which provides a summary of all WoKaS datasets attributes. The file includes information on the dataset’s country’s name, assigned WoKaS identifier, spring’s name, local gauge station identifier which is the assigned identifier in the database of origin of dataset, spring’s coordinates, spring’s discharge observation length (years), quality flags (as described in subsection accuracy and quality of datasets), dataset’s source name and the source type which indicates online or offline accessibility of the datasets (see subsection data acquisition and Table 1). Discharge observation measured at gauge stations located farther downstream of the spring’s outlet are usually influenced by superficial flow. When information about the influence of superficial flow is available, it is provided in the “Additional information” section of the metafile. A more comprehensive explanation of the used terms and content of the metafile is provided in the “read_me” document file included in the zip file archive.
(B) WoKaS_Hydrograph_Datasets includes over 400 CSV files of karst spring discharge measurements in cubic metres per second (m^3^/s). Headers providing meta-information such as the source of the dataset, spring’s name, local gauge station identifier, location coordinates in WGS 84 as well as measurement time format are appended to the csv files. The discharge observations provided in the data repository^23^ can be static or dynamic. Datasets obtained from research partners, collaborators and publications are static because they are not updated periodically. Conversely, datasets from hydrological databases (see subsection hydrological agencies) are dynamic and periodically updated through the individual agencies. For users who want the updated datasets, they can be obtained directly from the source online database via an automatic download routine (see below). As described in the Methods sections, for some sources (Banque Hydro, eHYD, LUBW and NRFA), complete datasets at higher temporal resolution are only obtainable through the automatic download routine (see information in “Additional information” column of the metafile).
(C) Auto_Download_Routine includes the R script files for downloading the dynamic datasets from the hydrological agencies online databases. The R codes allow the user to access and download the most recent version of the discharge datasets in their original temporal resolution from the online databases of the hydrological agencies. The downloaded datasets from the different online databases are standardised, having same format as described in “B” above. The newly downloaded version of the dynamic datasets are saved in WoKaS_Dynamic_Datasets folder, which is automatically created while the download routine code is run. In case of changes within the hydrological databases online access link system, the R codes might stop working. However, the R code will be frequently maintained and an updated version will be available on GitHub (https://github.com/KarstHub/WoKaS).
(D) WoKaS_Summary_Plot subfolder contains a pdf file also named “WoKaS_Summary_Plot which includes the hydrograph plots of all the spring discharge datasets. The name of each plot is the name of the corresponding dataset contained in the WoKaS_Hydrograph_Datasets subfolder.

### Datasets naming convention

The naming convention used for the datasets is a combination of the International Organisation for Standardisation Alpha-2 (ISO 2) country’s code, and a four-digit serial number followed by the spring’s name. The ISO 2 code and four-digit serial number are separated by a hyphen “-” and an “@” sign between the serial number and the spring’s name. For example, a dataset with the name “FR-0050@Cent-Fonts” means:

‘FR’ = ISO-2 country’s code for France

‘0050’ = WoKaS database assigned serial number

‘Cent-Fonts’ = Name of the spring.

### Recommended usage for datasets

Based on the assigned quality classes for the datasets, we provide recommendation on the usage and application of the datasets. The “very high” and “high” quality datasets (Class A and B) are appropriate for all hydrological analyses including statistical and comparative analyses, model evaluation and calibration, impact studies and process understanding. The C1 datasets are suitable for discharge’s trend analysis, event-based process understanding and water balance estimation. It should be noted that human impacts such as groundwater pumping for drinking and irrigation could affect spring discharge and trends can’t be solely attributed to climatic and landscape changes. In the comment section of the metafile, information about human impacts are only included when available. C2 and C3 quality datasets are most suitable for analysis that does not require much accuracy, such as computing annual averages or monthly spring discharge variations.

The focus of the WoKaS database is to provide easy access to spring discharge dataset, the present structure of the database does not distinguish among different aquifer recharge processes that fed the karst springs (see subsection accuracy of identified locations). Where autogenic recharge prevails, precipitation infiltrates directly into the aquifer through the carbonate rock surface. Whereas, allogenic recharge is due to inflows from non-carbonate units infiltrating into the aquifer through swallow holes, sinking streams, etc^9^. For users interested in distinguishing the recharge processes, recharge processes can be revealed through comparing carbonate rock outcrops and topographic catchment areas. A comprehensive water balance of the spring catchment area may reveal if the aquifer recharge is entirely feed from the carbonate area or if adjacent non-carbonate areas contribute water, as well. Furthermore, allogenic recharge is often associated with sinking streams and disappearing rivers, information on stream density and discontinuities of river networks^26^ can provide evidence of allogenic recharge.

### Outlook

Presently, access to WoKaS datasets is possible through the figshare repository^23^. In future, we hope that the database can be integrated into a web application platform for visualisation, further data uploads, and easier download.

## Data Availability

The R code to download datasets directly from the hydrological databases and to combine them with the spring discharge time series obtained from the other sources (see above) is available at https://github.com/KarstHub/WoKaS. The code is provided in R programming language version 3.5.0, and commented following a recommended programming comment guidelines^24^. Comprehensive instructions on how to run the code and system requirements are provided by a “README” file included in the GitHub repository.
