# Author Correction: Global karst springs hydrograph dataset for research and management of the world’s fastest-flowing groundwater

**DOI:** 10.1038/s41597-020-00590-3

**Published:** 2020-07-30

**Authors:** Tunde Olarinoye, Tom Gleeson, Vera Marx, Stefan Seeger, Rouhollah Adinehvand, Vincenzo Allocca, Bartolome Andreo, James Apaéstegui, Christophe Apolit, Bruno Arfib, Augusto Auler, Vincent Bailly-Comte, Juan Antonio Barberá, Christelle Batiot-Guilhe, Timothy Bechtel, Stephane Binet, Daniel Bittner, Matej Blatnik, Terry Bolger, Pascal Brunet, Jean-Baptiste Charlier, Zhao Chen, Gabriele Chiogna, Gemma Coxon, Pantaleone De Vita, Joanna Doummar, Jannis Epting, Perrine Fleury, Matthieu Fournier, Nico Goldscheider, John Gunn, Fang Guo, Jean Loup Guyot, Nicholas Howden, Peter Huggenberger, Brian Hunt, Pierre-Yves Jeannin, Guanghui Jiang, Greg Jones, Herve Jourde, Ivo Karmann, Oliver Koit, Jannes Kordilla, David Labat, Bernard Ladouche, Isabella Serena Liso, Zaihua Liu, Jean-Christophe Maréchal, Nicolas Massei, Naomi Mazzilli, Matías Mudarra, Mario Parise, Junbing Pu, Nataša Ravbar, Liz Hidalgo Sanchez, Antonio Santo, Martin Sauter, Jean-Luc Seidel, Vianney Sivelle, Rannveig Øvrevik Skoglund, Zoran Stevanovic, Cameron Wood, Stephen Worthington, Andreas Hartmann

**Affiliations:** 1 Chair of Hydrological Modeling and Water Resources, University of Freiburg, 79098 Freiburg, Germany; 2 Department of Civil Engineering, University of Victoria, Victoria, Canada; 3 Chair of Hydrology, University of Freiburg, 79098 Freiburg, Germany; 4 Department of Earth Sciences, Shiraz University, Shiraz, Iran; 5 Dipartimento di Scienze della Terra, dell’Ambiente e delle Risorse, University of Naples Federico II, Napoli, Italy; 6 Department of Geology and Centre of Hydrogeology of the University of Málaga, Málaga, Spain; 7 Instituto Geofísico del Perú, Lima, Peru; 8 Universidad Nacional Agraria La Molina, Maestria en Recursos Hídricos, Lima, Perú; 9 Parc Naturel Régional des Grands Causses (PRNGC), Saint-Léons, France; 10 Aix Marseille Univ, CNRS, IRD, INRA, Coll France, CEREGE, Aix-en-Provence, France; 11 Instituto do Carste/Carste Ciência e Meio Ambiente, Belo Horizonte, Brazil; 12 BRGM, Univ. Montpellier, Montpellier, France; 13 HydroSciences Montpellier (HSM), Univ. Montpellier, CNRS, IRD, Montpellier, France; 14 Earth and Environment, Franklin and Marshall College, Lancaster, Pennsylvania USA; 15 ISTO, Université d’Orléans, CNRS, BRGM, OSUC, Orléans, France; 16 Faculty of Civil, Geo and Environmental Engineering, Technical University of Munich, Arcisstr. 21, 80333 Munich, Germany; 17 ZRC SAZU Karst Research Institute, Postojna, Slovenia; 18 Cave and Karst Specialist, Vientiane, Laos; 19 BRGM, Univ. Montpellier, Montpellier, France; 20 Institute of Applied Geosciences, Karlsruhe Institute of Technology (KIT), Kaiserstr. 12, 76131 Karlsruhe, Germany; 21 Institute for Geography, University of Innsbruck, Innrain 52, Innsbruck, Austria; 22 School of Geographical Sciences, University of Bristol, Bristol, BS8 1SS UK; 23 Department of Geology, American University of Beirut, Beirut, Lebanon; 24 Applied and Environmental Geology, Department of Environmental Sciences, University of Basel, Bernoullistr. 32, 4056 Basel, Switzerland; 25 Normandie Univ, UNIROUEN, UNICAEN, CNRS, M2C, 76000 Rouen, France; 26 School of Geography, Earth and Environmental Science, University of Birmingham, Birmingham, UK; 27 Institute of Karst Geology, Chinese Academy of Geological Sciences, Guilin, China; 28 GET Laboratory, Toulouse University/CNRS/IRD, Toulouse, France; 29 Department of Civil Engineering, University of Bristol, Bristol, UK; 30 Barton Springs/Edward Aquifer Conservation District, Austin, Texas USA; 31 Institut Suisse de Spéléologie et de Karstologie, ISSKA, CH-, 2301 La Chaux-de-Fonds, Switzerland; 32 Department for Environment and Water, Government of South Australia, Adelaide, Australia; 33 University of São Paulo, São Paulo, Brazil; 34 Institute of Ecology, School of Natural Sciences and Health, Tallinn University, Uus-Sadama 5, 10120 Tallinn, Estonia; 35 Department of Applied Geology, Georg-August-University Göttingen, Goldschmidstr. 3, 37077 Göttingen, Germany; 36 Géosciences Environnement Toulouse (GET) - CNRS – UPS – IRD – CNES, 14 avenue Edouard Belin, 31400 Toulouse, France; 37 Department of Earth and Environmental Sciences, University Aldo Moro, Bari, Italy; 38 INRAE, Avignon Université, EMMAH, F-84000 Avignon, France; 39 LOCEAN Laboratory, Sorbonne-Université/CNRS/IRD/ MNHN, Paris, France; 40 University Federico II, Naples, Italy; 41 Department of Geography, University of Bergen, Bergen, Norway; 42 Faculty for Mining and Geology, University of Belgrade, Belgrade, Serbia; 43 Worthington Groundwater, Ontario, Canada

Correction to: *Scientific Data*10.1038/s41597-019-0346-5, published online 20 February 2020

The following authors were omitted from the original version of this Data Descriptor: Vincent Bailly-Comte, Perrine Fleury, Jean-Christophe Maréchal and Jean-Luc Seidel. The author list and affiliations have been amended to address these omissions in both the HTML and PDF versions.

